# Thrombotic Microangiopathy: A Devastating Complication After Lung Transplantation

**DOI:** 10.1097/TXD.0000000000001758

**Published:** 2025-03-10

**Authors:** Wouter Rosseels, Laurent Godinas, Papay Jallah, Robin Vos, Lieven Dupont, Dirk Kuypers, Thomas Vanhoutte, Kathleen J. Claes

**Affiliations:** 1 Department of Nephrology and Renal Transplantation, UZ Leuven, Leuven, Belgium.; 2 Department of Respiratory Diseases, UZ Leuven, Leuven, Belgium.; 3 Department of Microbiology, Immunology, and Transplantation, Nephrology and Renal Transplantation Research Group, KU Leuven, Leuven, Belgium.

## Abstract

**Background.:**

Thrombotic microangiopathy (TMA) following lung transplantation (LTx) is a rare but severe complication. The pathogenesis is poorly understood, and various risk factors have been suggested. In this study, we aimed to evaluate diagnostic accuracy, identify risk factors, and assess renal, pulmonary, and overall survival of TMA in this patient group.

**Methods.:**

We performed a case-control retrospective study of patients with TMA after LTX between January 1, 2000, and January 1, 2021. Controls were selected based on underlying lung disease, age, sex, cytomegalovirus risk, and immunosuppressive regimen. Overall survival data were collected for the whole lung transplant group.

**Results.:**

A total of 29 TMA cases (2.9%) were identified out of 1025 LTx. Median time to development of TMA was 5.9 mo, 76% occurred in the first 12 mo. In the TMA group a higher rate of HLA donor-specific antibodies (11% versus 1%; *P* = 0.05), a lower median time to onset of chronic lung allograft dysfunction (37 versus 91 mo; *P* = 0.0017), a higher rate of cytomegalovirus infection (45% versus 19%; *P* = 0.02), and a higher prevalence of end-stage renal disease (24% versus 6%; *P* = 0.03) and overall death (97% versus 44%; *P* < 0.0001) was found. Diagnostic assessment of TMA was complete in 48% of patients, with Coombs testing missing in 52% and a disintegrin and metalloproteinase with thrombospondin type 1 motif 13 activity not assessed in 59%.

**Conclusions.:**

TMA poses a significant risk of end-stage renal disease and mortality after LTx. Challenges remain in standardizing diagnostic criteria and understanding its pathogenesis, underscoring the need for unified protocols in diagnosis and standardized screening. This study identifies potential risk factors and temporal patterns for TMA occurrence, providing crucial insights for future treatment strategies.

Thrombotic microangiopathy (TMA) is characterized by the presence of a Coombs negative microangiopathic hemolytic anemia and thrombocytopenia with or without acute organ injury. It is a well-known complication after allogenic BMT and solid organ transplantation, particularly kidney transplantation.^1-3^ TMA occurring after lung transplantation (LTx) is a rare but deleterious complication. The incidence of LTx associated TMA (LTx-TMA) varies between 1.8% and 7%, but available data are scarce.^4,5^ Furthermore, the classic triad as described above is not always present. In a retrospective study by Hachem et al,^4^ only 25% of cases (n = 6) were found to have the classic triad. Interestingly, in a renal biopsy study after LTx in patients with cystic fibrosis and acute kidney injury (AKI; n = 15), TMA was found in 7 (46.7%) patients.^6^ All patients were hypertensive at the time of diagnosis, but none of them showed hematologic signs of intravascular hemolysis or thrombocytopenia. This is further corroborated by the study of Schwarz et al,^7^ in which the renal biopsy findings after nonrenal transplantation were studied. The prevalence of TMA was the highest in the liver (13%) and lung (14%) transplant group. In this study, only half of the patients had clinical and hematologic signs of TMA. This implicates that the actual prevalence may be even higher. The pathogenetic mechanisms of TMA after nonrenal transplantation are poorly understood but appear to be multifactorial, and although consensus has grown about the role of endothelial damage in its pathogenesis,^8^ little is known about the risk factors for this endothelial cell damage. Most patients with TMA post-solid organ transplantation are treated with calcineurin inhibitors (CNIs) probably contributing to this “endothelial damaging milieu.”^1,9^ In the cystic fibrosis biopsy study,^6^ CNI toxicity was found in all renal-limited TMA cases. However, other factors such as ischemia/reperfusion injury, antibody-mediated rejection, and viral infections, especially cytomegalovirus (CMV), might contribute as well.^10,11^

Other risk factors identified in small case studies are female sex and previous TMA.^12^ Identifying these risk factors may have a pivotal role in both prevention and adequate treatment of LTx-TMA. Current treatment is supportive, consisting of treatment of the underlying conditions (rejection, infection, and arterial hypertension) and change of the immunosuppressive regimen, mostly switching from a CNI to another class of immunosuppressant. Plasma exchange has been performed but is currently not recommended.^13^ Furthermore, little is known about the outcome of these patients and the impact of changing immunosuppressive therapy on the organ, in casu lung, function.

In this retrospective observational case-control study, we aimed to describe the diagnostic evaluation and risk factors for the development of TMA and to study renal and pulmonary outcomes, as well as overall survival in the setting of TMA post-LTx.

## MATERIAL AND METHODS

### Study Design

We performed a retrospective study of all patients who underwent an LTx between January 1, 2000, and January 1, 2021, at University Hospitals Leuven, a member of the European Reference Network for Rare Kidney and Lung Diseases (ERKNet-ERNLung). Patient follow-up is available till January 9, 2023.

We performed a search in the electronic medical record for the following items: lung transplant AND hemolysis OR TMA OR hemolytic uremic syndrome OR atypical hemolytic uremic syndrome OR hemolytic anemia OR thrombotic thrombocytopenic purpura (TTP). Next to this we performed a search for patients with serum haptoglobin levels <0.3 mg/dL and/or presence of schistocytes >5/1000 red blood cells.

In the following step, individual files were analyzed by 2 physicians (W.R., K.J.C.) to adjudicate patients based on the following inclusion criteria: age >18 y, single or bilateral LTx, diagnosis of TMA based on laboratory markers indicating TMA (de novo thrombocytopenia or a decrease of 25% of thrombocytes AND microangiopathic hemolytic anemia) with or without signs/symptoms of organ dysfunction (AKI, neurological symptoms, gastrointestinal symptoms) since a striking frequency of subclinical TMA after transplantation of other organs has recently been reported.^7^

Demographic, clinical, and biological characteristics were obtained from patients’ electronic charts.

For the hematologic assessment, 2 different subgroups were defined based on the available data: (a) patients with complete TMA evaluation (haptoglobin, Coombs test, schistocytes) and (b) patients without Coombs results available. Exclusion criteria were combined transplantation (heart-lung, kidney-lung, liver-lung), death within 1 mo after transplantation, positive Coombs test, and referrals from another center.

Given the possible mimicking of hemolysis under chronic therapy with dapsone for *Pneumocystis jirovecii* prophylaxis these patients were excluded unless proven TMA on biopsy was present. To differentiate TTP as a subtype of TMA, a disintegrin and metalloproteinase with thrombospondin type 1 motif 13 (ADAMTS-13) activity was assessed. If ADAMTS-13 activity was <10% the diagnosis of TTP was made. Diagnosis of diffuse intravascular coagulation (DIC) was made based on prothrombin time, activated partial thromboplastin time, and fibrinogen levels.

Kidney damage was defined as the presence of AKI, according to the Kidney Diseases Improving Global Outcomes guidelines.^14^ Gastrointestinal tract involvement as the presence of diarrhea, upper or lower gastrointestinal bleeding, and/or abdominal pain. Involvement of the central nervous system was defined as a new onset of epilepsy, altered consciousness by exclusion, and development of stroke or confusion.

CMV status was defined as high risk (donor positive/receptor negative), low risk (donor and receptor negative), and intermediate risk (donor positive/receptor positive or donor negative/receptor positive). CMV infection was defined as either CMV disease with organ involvement or CMV syndrome.^15^

Arterial hypertension throughout the data collection was defined as a blood pressure >140/90 mm Hg and subdivided into its different classes according to the latest guidelines.^16^

For the laboratory values (estimated glomerular filtration rate based on serum creatinine before and at the time of transplantation, blood platelets, haptoglobin, and schistocytes), the worst values were recorded.

Subsequently, matched control patients were manually selected from the total cohort based on underlying lung disease, age, sex, CMV risk category (donor and receptor positivity/negativity), and immunosuppressive regimen. Patients were individually matched for these parameters in a 1:2 ratio. Exclusion criteria for the matched control patients were combined transplantation (heart-lung, kidney-lung, liver-lung), death within 1 mo after transplantation, and referrals from another center.

For the total cohort of LTx performed between January 1, 2000, and January 1, 2021, data on age, sex, and mortality were available and extracted.

The study was conducted in accordance with the Declaration of Helsinki; the Belgian law related to experiments in humans dated May 7, 2004; and the General Data Protection Regulation 2016/679 and the Belgian law of July 30, 2018, regarding the protection of personal data.

The Ethical Committee of the University Hospitals Leuven approved the study (approval number S66887) and waived the need for an informed consent because of the retrospective study design. Patients were informed of the use of their data.

### Statistical Analysis

Patient demographics were reported using descriptive statistics. Continuous variables were expressed using the median and interquartile range values or expressed as means ± SDs depending on the data distribution. Categorical variables were expressed as counts and percentages in each category.

Differences between groups, if statistically feasible depending on the number of events, were assessed by Wilcoxon rank-sum tests or *t* tests for continuous variables. Chi-square tests were used for categorical variables. The Cox proportional hazard model was used to determine the association of the occurrence of TMA with overall survival; in this model, TMA was introduced as a time-dependent covariate to prevent immortal time bias. A *P* values of <0.05 were considered statistically significant. In the case of multiple comparisons, Dunn or Bonferroni correction was applied.

## RESULTS

### Baseline Characteristics

A total of 1139 LTx were performed in 1090 patients in the time period between January 1, 2000, and January 1, 2021. Seventy-four patients had a combined transplantation, and 40 patients were younger than 18 y. Using the search terms described in the methodology section, 276 unique patients were selected.

After further analysis of these individual patient files, 222 patients were excluded because they did not meet the criteria for TMA, 22 patients received combined transplantation and 3 patients died within 1 mo after transplantation.

Twenty-nine patients (2.8%) met the abovementioned inclusion criteria for TMA. None of these patients had a history of TMA before transplantation. The control group consisted of 54 patients. For 2 patients with TMA no matched controls were found based on the 5 matching criteria because of the rare underlying pulmonary diagnosis (ie, bronchiolitis obliterans and bronchiectasis).

Tables 1 and 2 depict diagnostic assessment of the TMA group and comparison between baseline characteristics between the TMA group and matched controls.

**TABLE 1.** Clinical and biochemical parameters at time of TMA

	Total	
Biochemical variables	
Hemoglobin, g/dL, n = 29, mean ± SD	6.9 ± 0.99
Platelets, n = 29, mean ± SD	64 ± 32.1
Serum creatinine, mg/dL, n = 29, mean ± SD	2.78 ± 1.66
Tacrolimus through level, μg/L, mean ± SD	12 ± 3.2
Cyclosporin through level, μg/L, median (IQR)	225 (180–225)
Biochemical assessment, % available	
Schistocytes	100%
Coombs negative	48%
ADAMTS-13	55%
Haptoglobin	97%
PT/INR	100%
aPTT	83%
Fibrinogen	72%

ADAMTS-13, a disintegrin and metalloproteinase with thrombospondin type 1 motif 13; aPTT, activated partial thromboplastin time; INR, international normalized ratio; IQR, interquartile range; PT, prothrombin time.

**TABLE 2.** Comparison of baseline characteristics between TMA group and matched controls

Baseline characteristics	TMA group	Matched controls	Significance (*P*, TMA vs control)
No. of subjects	29	54	
Age at time of transplantation, y, median (IQR)	59 (56–61))	58 (56–61)	0.7
Sex, n (%)			0.99
Female	18 (62)	34 (63)	
Male	11 (38)	20 (37)	
Pulmonary diagnosis, n (%)			0.7
Bronchiectasis	1 (3)	0 (0)	
Bronchiolitis obliterans	2 (7)	2 (4)	
Chronic obstructive pulmonary disease	19 (66)	38 (70)	
Cystic fibrosis	2 (7)	4 (7)	
Pulmonary fibrosis	5 (17)	10 (19)	
Pretransplantation data			
Mean eGFRcr at time of transplantation (CKD-EPI 2009), mL/min/1.73 m^2^, mean ± SD	93.34 ± 14.6	91.94 ± 14.6	0.9
Arterial hypertension, n (%)	7 (24)	26 (41)	0.4
Cardiovascular disease, n (%)	3 (11)	8 (15)	0.9
Presence HLA donor-specific antibodies, n (%)	3 (11)	1 (2)	**0.05**
Proteinuria, n (%)	4 (14)	5 (9)	0.5
Cytomegalovirus risk status, n (%)			0.97
Donor–/receptor–	7 (24)	14 (26)	
Donor+/receptor–	11 (38)	20 (37)	
Donor–/receptor+	9 (31)	16 (30)	
Donor+/receptor+	2 (7)	4 (7)	
Transplantation data, n (%)			
Bilateral lung transplantation	26 (90)	47 (87)	0.99
Treatment induction			0.6
Standard^*a*^	21 (72)	43 (80)	
No induction^*b*^	8 (28)	11 (20)	
Immunosuppressive regimen, n (%)			0.4
Tacrolimus/mycophenolate mofetil/steroids	16 (55)	30 (56)	
Tacrolimus/azathioprine/steroids	5 (17)	12 (22)	
Cyclosporine/azathioprine/steroids	8 (28)	12 (22)	
CMV infection (within 1 y after transplantation for control group), n (%)			**0.02**
Cytomegalovirus infection	13 (45)	10 (19)	
Outcome, n (%)			
End-stage renal disease	7 (24)	3 (6)	**0.03**
Rejection	7 (24)	21 (39)	0.2
Time to rejection, mo, median (IQR)	18 (12–53)	4 (1–28)	0.15
CLAD, n (%)	8 (28)	16 (30)	0.99
Time to CLAD, mo, median (IQR)	21 (16–65)–69)	97(58–127)	**0.0017**
Death, n (%)	28 (97)	24 (44)	**≤0.0001**
Time from transplantation to death, mo, median (IQR)	27 (14–78)	60 (21–103)	**0.05**
Time from TMA to death, mo, median (IQR)	14 (3–72)		

^*a*^Treatment induction consisted of ATGs derived from the serum of rabbits at a dose of 3 mg/kg for 3 d.

^*b*^No induction was given in case of a positive SPT before ATGs or when a patient was already under immunosuppressive therapy pretransplantation. Bold indicates denotes statistical significance (*P* ≤0.05).

ATG, anti-thymocyte globulin; CKD-EPI, Chronic Kidney Disease Epidemiology Collaboration; CLAD, chronic lung allograft dysfunction; CMV, cytomegalovirus; eGFRcr, estimated glomerular filtration rate based on serum creatinine; IQR, interquartile range; SPT, skin prick test; TMA, thrombotic microangiopathy.

The underlying pulmonary disease was chronic obstructive pulmonary disease in the majority (66%) of patients, followed by pulmonary fibrosis (17%). Cystic fibrosis (7%), bronchiolitis obliterans (7%), and bronchiectasis (3%) were rare indications. The immunosuppressive regimen initiated directly posttransplantation varied with 55% receiving tacrolimus/mycophenolate mofetil/steroids, 17% tacrolimus/azathioprine/steroids, and 27% cyclosporin/azathioprine/steroids or cyclosporin/steroids (n = 1).

In none of the patients, a mammalian target of rapamycin (mTOR) inhibitor was used as the initial immunosuppressive agent. Adequately controlled arterial hypertension before transplantation was observed in 24% of the patients, cardiovascular disease in 11%. The mean estimated glomerular filtration rate based on serum creatinine before transplantation was 93.3 mL/min/1.73 m^2^ (Chronic Kidney Disease Epidemiology Collaboration 2009) with presence of slight proteinuria, mainly established on urinary dipstick, in 14% of the patients.

### Diagnostic Assessment

In 48%, a complete hematologic assessment was performed; in the remaining 52%, the results of the Coombs test were not available. For 2 patients, no results for haptoglobin or the presence of schistocytes were available. In 6 patients (20%), no full assessment of DIC was performed. Values of ADAMTS-13 activity, for diagnosis of TTP, were present in 59% of cases.

Organ damage was present in 77% of patients with AKI as the main organ involved (72%), 27% had neurologic complications, and 7% had gastrointestinal complications. Twenty percent of patients had both AKI and neurological damage and in 7% combined AKI with gastrointestinal complications were seen. TMA occurred in all but one of the patients after their first LTx.

### TMA Episodes

The median time from LTx to development of TMA was 5.9 mo (range, 1 to 52 mo), with 5 patients (17%) being diagnosed with TMA during the first month after LTx. The majority (79%) developed TMA within the first year after LTx.

In 72% of cases, an active infection was present at the time of TMA diagnosis. An active CMV infection, based on blood polymerase chain reaction, was present in 45% of cases, with proven organ involvement in 70% of these. Only 1 patient had a primo-infection, while reactivation was diagnosed mainly (77%) in the high-risk group (ie, CMV donor+/receptor–). Concerning the other causes of infection, 24% had a mono- or poly-bacterial infection, 17% had a fungal infection, and 3% had a viral infection other than CMV infection. Biopsy-proven acute rejection was present in 7% of patients at the time of TMA diagnosis. Arterial hypertension grade I or II^16^ at the time of TMA diagnosis was found in 31%. One patient was diagnosed with TTP. In 1 patient, an increased prothrombin time, activated partial thromboplastin time, and decreased fibrinogen were detected indicating a possible DIC.

Of the 6 patients who developed TMA >1 y after transplantation, 2 patients had a *Clostridioides difficile* infection, 1 patient had malignant hypertension and 1 patient had the immunosuppressive treatment was switched to a combination of tacrolimus and sirolimus 6 mo before the TMA episode because of an acute rejection. In the 2 remaining patients, an invasive pulmonary aspergillosis and a viral infection was diagnosed. In none of these patients, an active CMV infection was present. In 3 patients, TMA occurred in the immediate postoperative period before the introduction of CNI.

When we limited the analysis to the patients with a diagnosis between 1 and 12 mo after transplantation (n = 20), CMV infection was present in 65%, and in 5 patients, no active infections were identified. In 1 patient, TMA occurred after severe surgery (hemihepatectomy). In this case, ADAMTS-13 activity was undetectable leading to the diagnosis of TTP.

Therapeutic interventions, other than treating the underlying infection, included the following: most TMA patients (76%) had their immunosuppressive regimen changed, and 41% received plasmapheresis or plasma infusion. Two patients were treated with rituximab. Changes in immunosuppressive regimen were as follows: in 52%, tacrolimus was switched to cyclosporin, while in 7%, the reverse switch from cyclosporin to tacrolimus was made. In another 7%, tacrolimus was switched to everolimus in a CNI-free regimen. The dose of corticosteroids was increased in 2 patients and previously stopped corticosteroids were reintroduced in 1 patient. In rare cases, the target level for cyclosporin was lowered (3%) or treatment with tacrolimus was temporarily ceased (3%) without additional changes in immunosuppressive therapy.

These interventions led to a complete remission of TMA in 93% of all patients. Two patients died during their episode of TMA, which was complicated with a respiratory infection in both cases.

Recurrence of TMA after initial remission was observed in 2 patients. Complement genetic testing was performed in 4 patients, all of whom had negative results. In 2 of these patients, the testing was performed because of persistent or recurrent TMA.

### Matched Control and Total Patient Population Analysis

Table 2 highlights the demographic and clinical characteristics of the study cohort and provides an overview of the outcomes observed in both the TMA group and the matched controls.

When comparing the TMA group with the matched control group, there were no significant differences in age, sex, CMV status at the time of transplantation, and immunosuppressive regimen (as per protocol).

The only significant difference found between the 2 groups was a higher incidence of CMV infection and the presence of HLA donor-specific antibody (DSA; class II in 2 patients and combined classes I and II in 1 patient) at the time of transplantation in the TMA group. There was a trend towards a higher median age at time of LTx in the TMA group in comparison with the total cohort of LTx performed between January 1, 2000, and January 1, 2021 (n = 1025; *P* = 0.1) and a higher female predominance (*P* = 0.18).

### Outcomes

Four patients died within 3 mo after the occurrence of TMA, with ongoing TMA in 3 of them. Causes of death were CMV infection (n = 2), invasive pulmonary aspergillosis (n = 1), and chronic lung allograft dysfunction (CLAD) type restrictive allograft syndrome (n = 1). Two patients remained dialysis-dependent after their TMA episode.

**FIGURE 1.**
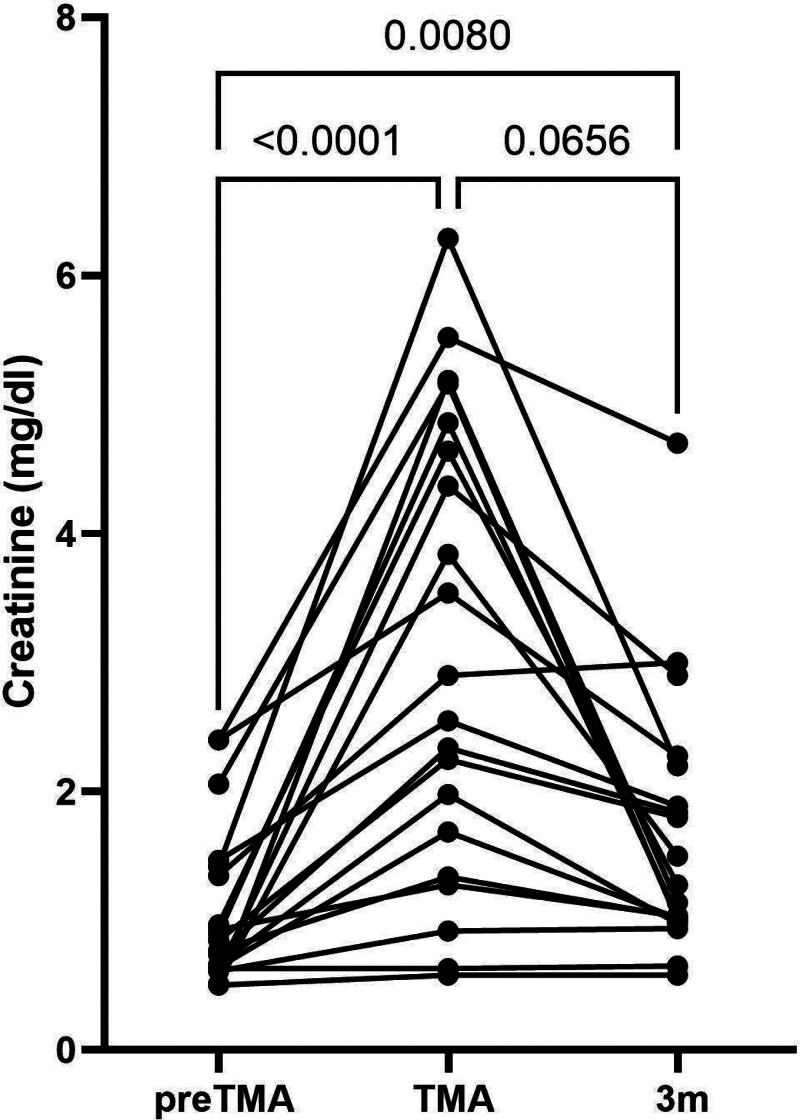
Evolution of serum creatinine before and at 3 mo after the TMA in nondialysis-dependent surviving patients. TMA, thrombotic microangiopathy.

Figure 1 demonstrates the evolution of serum creatinine before and at 3 mo after the TMA episode in the nondialysis-dependent surviving patients.

Twenty-four percent of patients evolved to end-stage renal disease (ESRD), an equal number experienced at least 1 rejection episode, and 28% developed CLAD. When compared with the control group, there was no difference in the occurrence of rejection or CLAD, but the time to onset of CLAD was shorter in the TMA group. Patients who developed TMA were at higher risk of ESRD (*P* = 0.03) or death (*P* < 0.0001). Causes of death in the TMA group were infection (50%), chronic graft dysfunction (29%), and malignancy (11%). In 7%, palliative care was initiated. In 1 patient, the cause of death was unknown.

**FIGURE 2.**
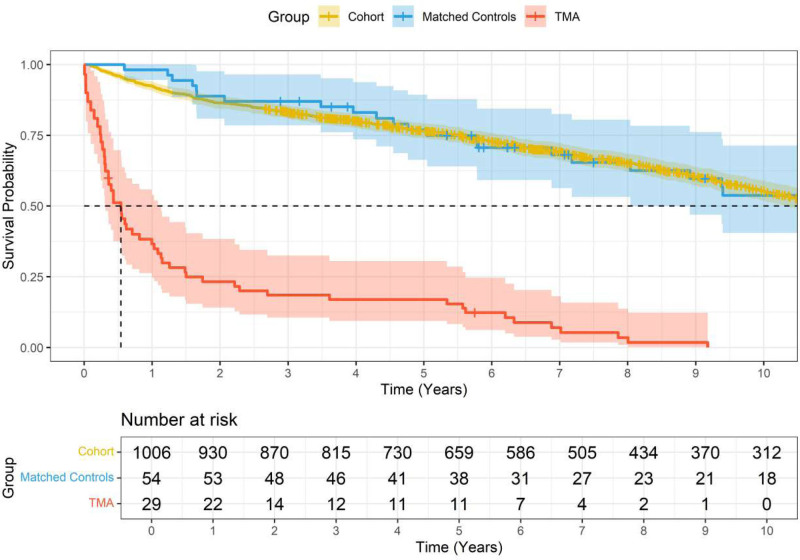
Overall survival in TMA patients, controls, and the total cohort of lung transplant patients with TMA as a time-dependent covariate. TMA, thrombotic microangiopathy.

Figure 2 illustrates overall survival in TMA patients (n = 29), controls (n = 54), and the total cohort (n = 1006) of LTx with TMA as time-dependent covariate.

Table 3 shows the association of TMA with all-cause death using data available for the total cohort in a multivariate Cox proportional hazards model adjusted for age and sex. While age (per year increase) and TMA status were highly significant independent predictors of overall survival, no significant association was found for sex.

**TABLE 3.** Multivariate Cox proportional hazards model

Covariate	HR	Lower_95	Upper_95	*P*
TMA	4.34	2.76	6.83	<0.0001
Age (per year)	1.04	1.03	1.05	<0.0001
Sex	1.06	0.89	1.25	0.52

HR, hazard ratio; TMA, thrombotic microangiopathy.

## DISCUSSION

Our study demonstrates that TMA after LTx is a rare but deleterious complication with a high risk for the development of ESRD and mortality. This study is the largest reported to date in this specific group of LTx recipients. Furthermore, we found distinct differences in the underlying risk factors depending on the time of diagnosis of the TMA event providing insight into the cause and potential future treatment of the disease. The median time from LTx to TMA development and the high percentage of cases within the first year after transplantation highlight the importance of vigilant monitoring and screening for TMA during this early posttransplant period.

We found an incidence of 2.8%, consistent with previously reported incidences.^4,5^ However, in LTx, data are limited to case series and 1 retrospective analysis by Hachem et al^4^ in a smaller group (n = 240) of patients. In our cohort, a full diagnostic assessment was frequently lacking illustrating the need for more awareness and highlighting even more the difficulty of diagnosing this disease entity because of lack of good diagnostic criteria and specific diagnostic biomarkers. Furthermore, given the high mortality as demonstrated in our study increased attention for this complication after transplantation is needed. Therefore, we urge the Lung Transplantation Society to harmonize definitions for screening and diagnostic criteria analogous to the recently published guidelines on TMA occurring after hematopoietic cell transplantation.^17^ In these guidelines, biochemical screening is recommended depending on timing after transplantation or concurrent events such as graft versus host disease. The screening tests consist of blood counts, blood pressure, lactate dehydrogenase measurements, schistocytes, and random urinary protein to creatinine ratio. In case of positive screen, further diagnostic evaluation is recommended to establish the underlying etiology (soluble complement 5b-9, ADAMTS-13 activity, Coombs, DIC panel, haptoglobin, and evaluation of end-organ damage). Based on our findings in this study, we propose to assess hemolysis parameters (lactate dehydrogenase, haptoglobin, schistocytes) and estimate proteinuria (random urinary protein to creatinine ratio) at the time of occurrence of CMV disease or syndrome, or in case of new onset of thrombocytopenia or a decrease of 25% in blood platelet count in LTx patients. In case of a positive screen, further differential diagnostics should be assessed as outlined in the transplantation-associated TMA recommendations. Further research, however, is warranted to establish the diagnostic role of soluble complement 5b-9 in this patient population.

Several risk factors have been suggested in previous case series in nonrenal transplant recipients: female sex, CMV infection, and the use of CNI and mTOR inhibitors. The potential role of CMV in the pathogenesis of TMA remains intriguing, with multiple single case reports identifying a potential association between CMV infection and TMA, mainly in HIV-infected patients. In transplant recipients, data are conflicting.^18,19^ Indeed, CMV can directly damage endothelial cells and can cause platelet adhesion to the microvascular wall by inducing the expression of endothelial adhesion molecules and the release of von Willebrand factor.^20^

However, the presence of other possible strong risk factors (eg, CNI, arterial hypertension, etc) for the development of TMA in transplant recipients hinders the possibility of making a firm conclusion about causality. Interestingly, we found distinct triggers depending on the timing of TMA, with a strikingly higher prevalence of CMV infection if TMA was diagnosed in the first year after transplantation, which may support a potential causal role. mTOR inhibition has also been suggested as a culprit for the development of TMA.^4,12^ In our series, the use of mTOR inhibitors was too low to draw any conclusion. In 1 patient, TMA occurred after switching from CNI to a mTOR inhibitor.

The identification of DSA being significantly higher in the TMA group at the time of transplantation raises questions about the potential role of an immunologic factor in TMA development. DSA binding anti-HLA class II complex has been shown to be able to bind to activated endothelial cells and trigger pulmonary vasculitis.^21^ However, recent studies have also demonstrated the occurrence of DSA-negative endothelial activation/vasculitis in LTx mediated by natural killer (NK) cells, possibly explaining TMA occurrence in a subset of patients.^22^ Interestingly, CMV infection is known to induce adaptive NK cell responses/changes in NK cell repertoire,^23^ it is therefore tempting to hypothesize that the higher incidence of CMV infection in the TMA group might have led to more NK cell activation, more extensive endothelial injury, and thus more aggressive rejection phenotypes, that is, a shorter time to the onset of CLAD, and complicated with TMA. Further exploring the interplay between viral, immunologic factors, endothelial damage, and TMA could provide crucial insights into the underlying mechanisms.

Current treatment of TMA after LTx consists of treatment of the underlying provoking condition and/or change of the immunosuppressive therapy. Plasma exchange is currently not recommended^13^ but, as demonstrated here, is still widely used in clinical practice. Little is known about the role of complement and its inhibition in this setting. We performed genetic analysis in a minority of patients (n = 4) and found no abnormalities consistent with previous studies where the frequency of finding a complement abnormality in secondary TMA was similar to healthy controls.^24,25^ Evidence supporting the use of eculizumab in this condition is limited. Case series demonstrate conflicting results.^24-27^ Unfortunately, a phase 3 trial investigating the efficacy and safety of ravulizumab (Alexion Pharmaceuticals, Boston, MA), a long-acting complement C5 inhibitor, in participants with secondary TMA has been terminated prematurely.

In addition, we demonstrated that despite the resolution of TMA in the vast majority of cases by changing their immunosuppressive regimen and/or treatment of the infection, 28 of the 29 TMA patients died during follow-up. Moreover, the time to CLAD was shorter in patients with TMA, possibly because of the changes in immunosuppressive regimen. Other factors, such as the high frequency of DSA in this population,^28^ may play a role. This is important as CLAD is the major cause of death after LTx,^29^ and future studies deciphering the possible link between TMA and CLAD are warranted.

This study also has its limitations. First, the retrospective design with the need for presence of hematologic signs of TMA implicates that underdiagnosis is definitively present. Furthermore, only in a limited number of patients, a renal biopsy was performed for confirming the TMA diagnosis. The study of Schwarz et al,^7^ in which renal biopsy findings after nonrenal transplantation were studied, indicated a prevalence of TMA of 14% after the LTx group while only half of the patients had clinical and hematologic signs of TMA raising the suggestion to lower the threshold for a kidney biopsy after nonrenal transplantation. Third, given the low patient number, we cannot definitively establish causality between TMA and reduced survival especially since CMV disease, and in particular CMV pneumonitis, has been associated with loss of graft function and survival in LTx recipients.^30^ Finally, as the control group was matched for immunosuppressive regimen, no firm conclusion can be drawn on the influence of immunosuppressive regimen on the development of TMA post-LTx.

In conclusion, this retrospective observational case-control study shows that despite being a rare condition, LTx-TMA poses a significant clinical diagnostic and therapeutic challenge with a high risk of morbidity (development of ESRD and faster occurrence of CLAD) and mortality. Our study emphasizes the critical need for continued research to unravel the intricate mechanisms of TMA in lung transplant recipients, aiming for improved diagnostic, preventive and therapeutic strategies to mitigate the impact of this complication on patient outcomes.
